# Hyperspectral Sensing of Disease Stress in the Caribbean Reef-Building Coral, *Orbicella faveolata* - Perspectives for the Field of Coral Disease Monitoring

**DOI:** 10.1371/journal.pone.0081478

**Published:** 2013-12-04

**Authors:** David A. Anderson, Roy A. Armstrong, Ernesto Weil

**Affiliations:** Department of Marine Sciences, University of Puerto Rico, Mayagüez, Mayagüez, Puerto Rico; U.S. Geological Survey, United States of America

## Abstract

The effectiveness of management plans developed for responding to coral disease outbreaks is limited due to the lack of rapid methods of disease diagnosis. In order to fulfill current management guidelines for responding to coral disease outbreaks, alternative methods that significantly reduce response time must be developed. Hyperspectral sensing has been used by various groups to characterize the spectral signatures unique to asymptomatic and bleached corals. The 2010 combined bleaching and Caribbean yellow band disease outbreak in Puerto Rico provided a unique opportunity to investigate the spectral signatures associated with bleached and Caribbean yellow band-diseased colonies of *Orbicella faveolata* for the first time. Using derivative and cluster analyses of hyperspectral reflectance data, the present study demonstrates the proof of concept that spectral signatures can be used to differentiate between coral disease states. This method enhanced predominant visual methods of diagnosis by distinguishing between different asymptomatic conditions that are identical in field observations and photographic records. The ability to identify disease-affected tissue before lesions become visible could greatly reduce response times to coral disease outbreaks in monitoring efforts. Finally, spectral signatures associated with the poorly understood Caribbean yellow band disease are presented to guide future research on the role of pigments in the etiology.

## Introduction

Coral diseases have significantly contributed to the loss of coral cover and diversity on tropical coral reefs around the world. Management plans for monitoring and responding to coral bleaching and disease outbreaks have been developed by various academic and governmental organizations [1]–[3]. The main strategies introduced by these plans are continual visual monitoring of protected reefs by marine park staff and volunteers, the use of remote sensing data to assess outbreak risk, and some form of response and decision making process to minimize coral mortalities [4]–[7]. The general strategies established by management plans require validation of the onset of an outbreak and causative agent. Therefore, diagnostic tools are required before preventative management decisions can be made.

Diagnosis of the most virulent coral diseases depends on the observation of lesions and tissue mortality in the field [8]. These events are rarely preceded by visual symptoms that provide an advanced alert of an increased risk of disease outbreaks. To that end, investigations of the cellular and molecular stress responses of corals and symbionts have aided in the development of models of coral stress physiology (Table 1) [9]–[24]. In general, the regulation of antioxidants, reactive oxygen species, and apoptosis underpin host homeostasis. From these models, molecular biomarkers of coral stress have been developed and promoted as necessary tools in coral disease diagnosis [25]. Despite these advancements, however, biomarkers of coral stress have not been integrated into long-term monitoring efforts [26]–[27].

**Table 1:** Molecular and cellular investigations with applications to coral health diagnostics.

Reference	Stress	Marker Discovered
[9]	Pathogen	Peroxidase
[10]	Pathogen	Programmed cell death gene expression
[11]	Pathogen	Mannose-binding lectin gene expression
[12]	Pathogen	FPs*
[13]	Pathogen	Antimicrobial gene expression
[14]	Thermal	General stress gene expression
[15]	Thermal	FPs gene expression*
[16]	Thermal	Apoptosis, symbiont density
[17]	Thermal	General stress gene expression
[18]	Thermal	Endosymbiont pigments*
[19]	Thermal and Light	Heat shock protein, actin gene expression
[20]	Thermal and Pathogen	Cell Counts, melanin*
[21]	Thermal and Pathogen	PPO, bactericidal activity, and lysozymes*
[22]	Thermal and Pathogen	PPO, bactericidal activity, antioxidants, FPs*
[23]	Unknown	PPO, melanin, FPs*
[24]	Unknown	PPO and immune cell counts*

Studies that that revealed pigment biomarkers are denoted by *. Prophenoloxidase and fluorescent proteins are abbreviated by PPO and FPs, respectively.

The use of hyperspectral sensing for the diagnosis of ecological diseases is a tool that is widely used in the fields of agriculture and forestry [28]–[35]. One of the proven benefits of this method is the ability to detect spectral changes before visual symptoms become apparent. In these investigations a combination of multivariate statistical analyses (e.g. cluster, principal component, discriminant function), data transformations (e.g. normalizations, derivative spectra), and spectral indices (e.g. sum of derivative spectra) are used to find the optimal set of methods for differentiating between disease states. Similar methods have been applied to the characterization of the spectral reflectance patterns of healthy and stressed corals [36]–[40]. However, the diagnosis of infectious coral disease using hyperspectral sensing has not been reported to date. In the present study, it was hypothesized that hyperspectral remote sensing could be used to distinguish between disease states that are indistinguishable through visual examination in the field.

To successfully diagnose ecological diseases using hyperspectral reflectance measurements, an understanding of the physiological mechanisms that control pigment concentrations is not required [31]. However, one of the potential benefits of using this method is the ability to link pigment compositions of the host, symbiont and pathogen to spectral signatures that are characteristic of each disease state. Derivative analysis of hyperspectral data has been used previously to model pigments and spectral features of the coral holobiont [36]–[40]. This is made possible, in part, by an extensive knowledge base on the absorption spectra of pigments in corals and their associated microbiota (Table 2). Additionally, the literature on molecular and cellular biomarkers of coral health, summarized in Table 1, has shown that changes in coral and symbiont pigment concentrations can be correlated with levels of disease stress. Such studies have used this data to infer the physiological roles that pigments play in coral disease etiology [9], [11], [18]–[19]. To that end, the present investigation analyzed whether hyperspectral reflectance data of diseased and healthy corals could be used to detect pigments that are known to be associated with coral disease. Detection of such pigments would provide evidence that *in vivo* spectroscopy is a feasible method of monitoring coral health status.

**Table 2:** Summary of hyperspectral sensing investigations, including the experimental variable studied and the spectral markers identified.

Reference	Experimental Variable	Marker Discovered
[37]	Benthic competition	Pigmentation
[38]	Bleaching	Overall absorbance
[39]	Bleaching	Overall fluorescence
[40]	Bleaching	Chlorophyll pigment density
[41]	Light intensity	Green and red fluorescence
[42]	Mortality	Overall reflectance
[43]	Mortality	Remote sensing reflectance
[44]	Natural variability	Overall reflectance
[45]	Salinity	Overall reflectance
[46]	Species	Overall reflectance
[47]	Species	Overall reflectance
[48]	Species	Overall fluorescence
[49]	Species and color morph	Overall reflectance
[50]	Species and geographic location	Overall reflectance
[51]	Time of day	Overall absorbance

In the last two decades, one of the major reef-building species of Caribbean coral reefs, *Orbicella faveolata* (formerly classified as *Montastraea faveolata*) [52], has suffered significant declines in coral cover and abundance in many localities due to a combination of bleaching, disease outbreaks, and anthropogenic stressors [53]–[54]. While this species is susceptible to at least eight different biotic and environmentally-driven disease conditions, it is still abundant and showing signs of recovery without the reappearance of disease symptoms in many affected localities [55]–[56]. However, diseases that are linked with climate warming, such as Caribbean yellow band disease (CYBD) and bleaching (BLE), continue to affect populations [57]–[60]. Cellular and molecular research on the recent decline of *O. faveolata* populations has transformed this species into a model of coral stress and disease physiology [21], [25], [61]–[64].

The primary objective of the present study was to provide proof of concept that hyperspectral sensing can be used to diagnose coral disease states. Results from cluster analysis and spectral indices demonstrate that *in vivo* spectroscopy can be used to diagnose disease states by separating CYBD, BLE and asymptomatic (ASYM) samples of *O. faveolata* into discrete groups. Derivative analysis highlighted subtle spectral features putatively associated with coral host and symbiont pigments. This allowed for the development of a framework for future research on the role that pigments play in the etiology of coral disease. Although the present study used samples measured *ex situ* with a hyperspectral radiometer, it is suggested that this basic spectroscopic method can be adapted for use *in situ*. Most notably, the results reported here have direct and immediate applications to a number of activities that are threatened by coral diseases, such as coral mariculture, reef restoration, and programs that use collections of living corals in aquaria to promote coral reef conservation [65]–[69].

## Methods

To investigate the effects of thermal stress-induced bleaching and Caribbean yellow band disease (CYBD) on reflectance spectra, we sampled wild colonies of *Orbicella faveolata* with and without visible symptoms of bleaching and disease for *ex situ* hyperspectral reflectance measurements. Samples were collected under a general collecting permit issued by the Department of Natural Resources of Puerto Rico. *Orbicella faveolata* is not a protected species under the US endangered and threatened species act.

### Sample Collection

**Figure 1:**
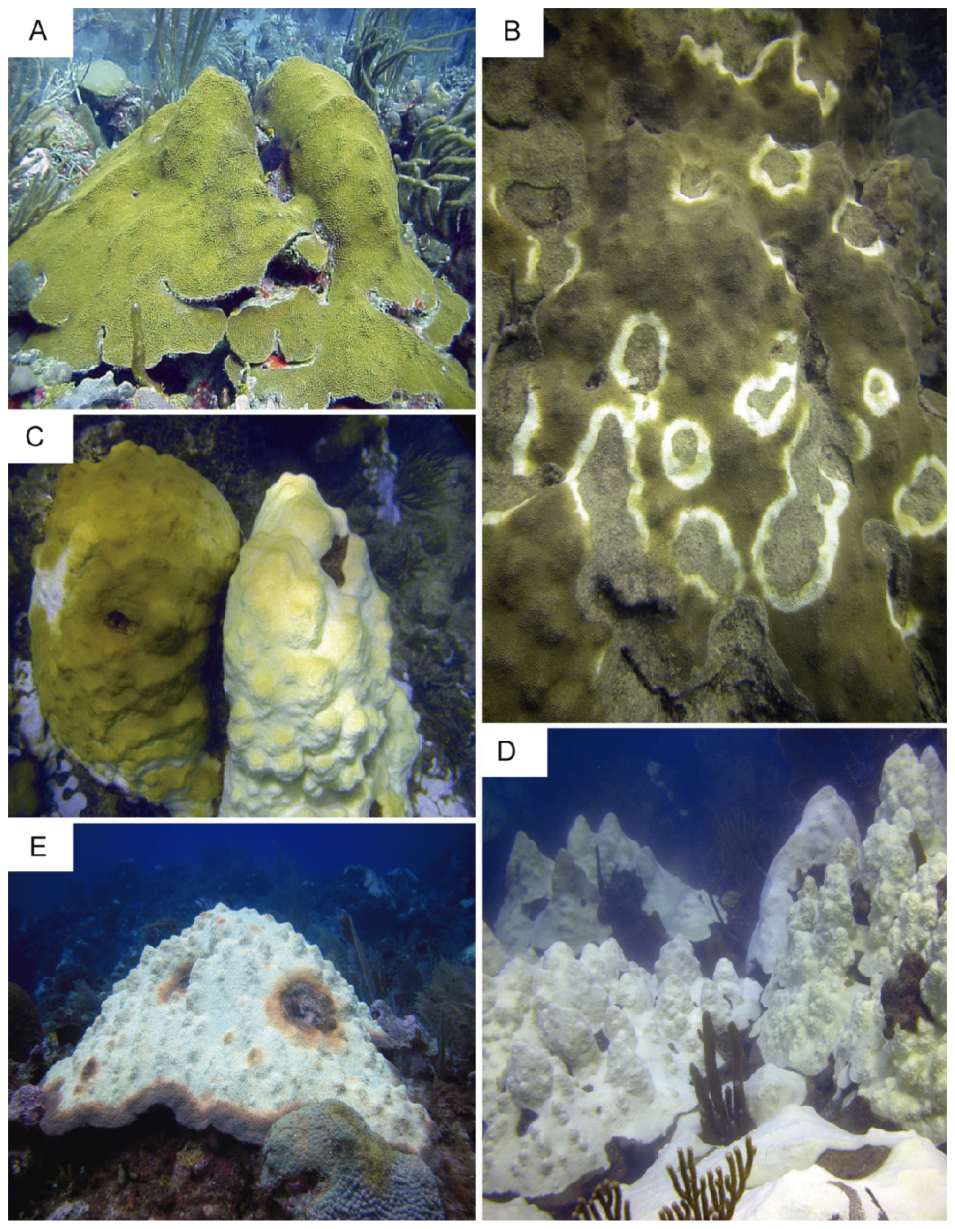
*Orbicella faveolata* with different health conditions at the study site in Puerto Rico. (A) An asymptomatic colony of *O. faveolata*, (B) a large colony with multiple focal infections of Caribbean yellow band disease, (C) bleached and unbleached colonies side by side, (D) several medium-sized bleached colonies, and (E) a large bleached colony with signs of Caribbean yellow band disease (Photos by E. Weil).

Sample collection took place during a single 45 min dive in October 2010, during a thermal anomaly and bleaching event. Ten degree-heating weeks were reported for this region on the date of sample collection (NOAA/NESDIS data, http://www.ospo.noaa.gov/data/cb/dhw/2010/dhwa.10.4.2010.gif). Degree-heating week is a metric developed by Gleeson and Strong [70], which is used as a remote sensing quantification of accumulated thermal stress in corals [70]–[72]. Samples (25 cm^2^) were collected at a depth of 10 m on the Media Luna fringing reef, (17° 56.091 N, 67° 02.577 W) in La Parguera, on the Southwestern coast of Puerto Rico. Photographic examples of each disease condition are presented in Figure 1. Sample collection was carried out carefully by fragmenting small portions of coral colonies using a hammer and sharp chisel. Colonies exhibiting signs of complete thermal bleaching (BLE, n = 5) were sampled (Figure 1C, E). Samples were also collected from lesions (CYBDL, n = 5) and asymptomatic regions of the same colonies (CYBDA, n = 5) (Figure 1B). CYBD colonies that were sampled did not exhibit visual symptoms of bleaching (Figure 1D). CYBDA samples were collected at a minimum distance of 20 cm from the lesion. Control colonies, referred to as asymptomatic (ASYM, n = 10), that did not exhibit visible signs of bleaching or disease were also sampled (Figure 1A). It is worth noting that CYBDA tissue and ASYM tissue are indistinguishable by visual observations in the field. Once collected, samples were shaded from direct sunlight, transported to the laboratory at ambient sea water temperature, and allowed to acclimate for 24 hours at the Department of Marine Sciences of the University of Puerto Rico field station in Isla Magueyes. The average laboratory conditions were 27.7°C for atmospheric temperature, 29.7°C for seawater temperature, and 33.6 practical salinity. During this acclimation period, samples were held in water tables and maintained at ambient temperatures using seawater pumped directly from within 1 km of the sampling site. This water was sand-filtered to reduce effects of sediment and organic particle loading. Water tables were shaded from direct sunlight to prevent light stress. Weather conditions during the acclimation and experimental measurements were overcast without precipitation, further reducing the chances of stress due to overexposure to direct sunlight or fluctuations in salinity.

### Hyperspectral Remote Sensing

After 24 hours of acclimation, the water level above the samples was reduced to 10 cm to reduce light attenuation, and the shades were removed for hyperspectral reflectance measurements under ambient light. A portable spectroradiometer (GER 1500) with a fiber optic cable was used to make two measurements in series of the upwelling radiance. Duplicate measurements were sufficient to minimize noise in the reflectance data. However, under different lighting conditions a greater number of measurements may be required. Measurements were made below the water surface at 3 cm from the sample tissue. The duplicate measurements were later averaged. The GER 1500 has a spectral range of 350 nm to 1050 nm with 512 channels and a spectral resolution of 2.8 nm. Percent reflectance was calculated according to methods established in the field of remote sensing [73]. Measurements were calibrated using a 50% reflectance Spectralon reference panel.

### Data Analysis

The magnitude and variability of the reflectance spectra for each colony condition was investigated for gross differences. Since the spectral data did not fit the assumptions for parametric analyses, a non-parametric Kruskal-Wallis (KW) test was used to determine the significance of colony condition on the magnitude of median reflectance within the visible spectrum. To assess the degree to which colony condition can be separated by the hyperspectral reflectance data within the visible wavelengths (400–680 nm), weighted average linkage cluster analysis was performed. This method has been used in various hyperspectral reflectance investigations [35]–[36].

Reflectance data were smoothed according to the Savitsky-Golay method [74], which has been previously established in the field of coral hyperspectral sensing [73]. Derivatives of the fourth order were used to identify spectral signatures that contribute to the shape of the reflectance spectra [75]–[76]. The position of derivative peaks with significant differences between disease conditions were compared to absorption peaks of known coral and symbiont pigments. The median magnitude of the derivative peaks were used in KW and Mann Whitney (MW) pairwise comparisons. In addition, various remote sensing indices commonly used in agricultural and forestry were tested for their ability to separate colony conditions [77]. Results are presented for the shift in the position of the red edge around 700 nm, and the sum of the first derivatives of the blue edge region (SD_B_) between 490 and 530 nm. The detection of the red edge shift was made possible by reducing the water level above the samples to minimize red light attenuation by the water column.

## Results

**Figure 2:**
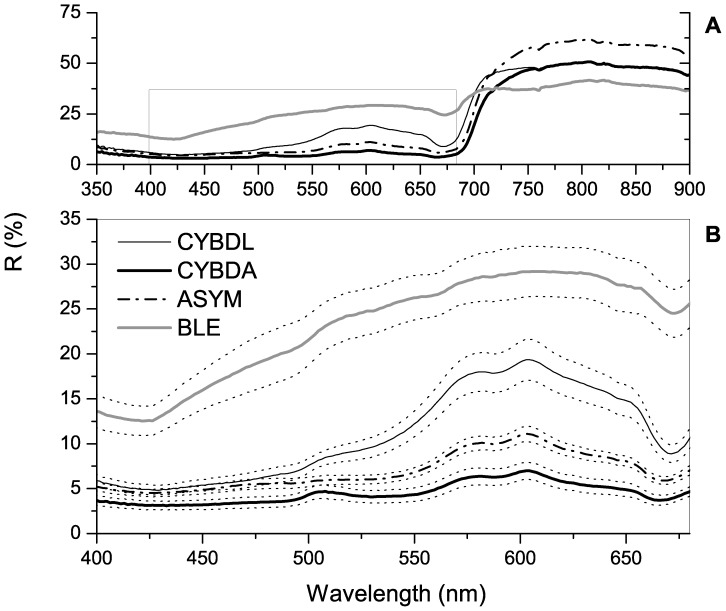
Percent reflectance (%R) for each condition. Caribbean yellow band disease lesion (CYBDL) and asymptomatic tissue (CYBDA), asymptomatic (ASYM), and bleached (BLE) sample averages. (A) %R of condition averages from 350 to 900 nm with selection of spectra presented in (B). (B) %R of condition averages from 400 to 680 nm with (+/−) standard errors represented by dotted lines, with significant differences demonstrated by Kruskal Wallis analysis (*H>14.00*, p<.005) between 420 and 660 nm.

**Figure 3:**
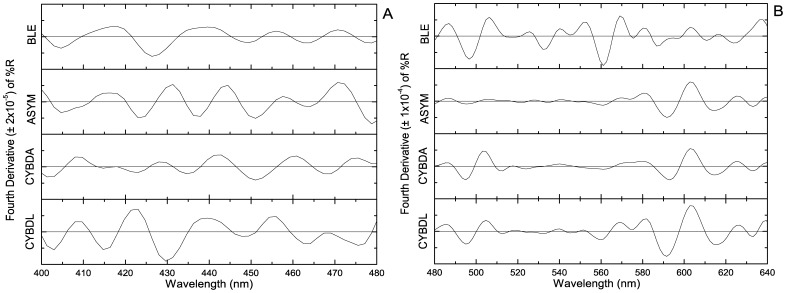
Average fourth derivative analysis of reflectance bands. (A) Between 400 and 480 nm, (B) between 480 and 630 nm for each colony condition. Caribbean yellow band disease lesion (CYBDL) and asymptomatic tissue (CYBDA), asymptomatic (ASYM), and bleached (BLE) sample averages.

Average remote sensing reflectance for each colony condition demonstrated the typical profile for tropical reef-building corals (Figure 2A). The albedo within the visible spectrum from greatest to least for each colony condition was BLE, CYBDL, ASYM, and CYBDA, respectively (Figure 2B). Significant differences in the magnitude of median reflectance were demonstrated for all wavelengths between 420 and 660 nm by KW analysis (H>14.00, p<.005). The standard error curves demonstrate low variability within colony conditions. The objective of the derivative analysis was to detect subtle spectral differences between colony conditions. Positive peaks in fourth derivative analyses (Figure 3A, B) that demonstrated significant differences in MW pairwise condition comparisons correspond to wavelengths that contribute to spectral differences between conditions. The results for these comparisons are reported in Table 3 along with pigments that putatively contribute to reflectance and absorbance at those wavelengths.

**Table 3:** Putative pigments that contribute to significant differences in derivative spectral signatures.

Wavelength (nm)	ASYM	CYBDA	CYBDL	BLE	p-value	Putative Pigment
423	A	A	B	A	<0.05	α-Carotenoid
428	A	A	B	B	<0.01	β-Carotenoid
436	A	A	B	B	<0.05	Chlorophyll-a
443	A	A	AB	B	<0.05	α-Carotenoid, Xanthophyll
455	A	A	B	B	<0.05	Xanthophyll
471	A	AB	B	C	<0.05	α-Carotenoid
476	A	B	A	A	<0.05	Xanthophyll, Peridinin
487	A	A	B	B	<0.05	Phycobilins
505	A	B	B	B	<0.005	GFP
526	A	A	A	B	<0.05	Fucoxanthin, Diadinoxanthin
540	A	A	A	B	<0.05	Fucoxanthin, Peridinin
553	A	AB	B	C	<0.05	Fucoxanthin
572	A	AB	C	B	<0.05	Red fluorescent protein
617	A	A	A	B	<0.01	Phycocyanin, Chlorophyll-a
626	A	A	A	B	<0.05	Phycocyanin, Chlorophyll-a
636	A	A	A	B	<0.01	Phycocyanin,
640	A	A	B	A	<0.01	Phycocyanin,

Wavelengths are reported where significant effects (p-value<0.05) of colony condition are demonstrated by Kruskal Wallis analysis of variance. Results of Mann Whitney pairwise comparisons demonstrate significant differences between colony conditions that have different letters. Putative pigments associated with each wavelength are suggested based on present knowledge of coral and symbiont pigment absorbance or reflectance [19], [44], [76].

**Figure 4:**
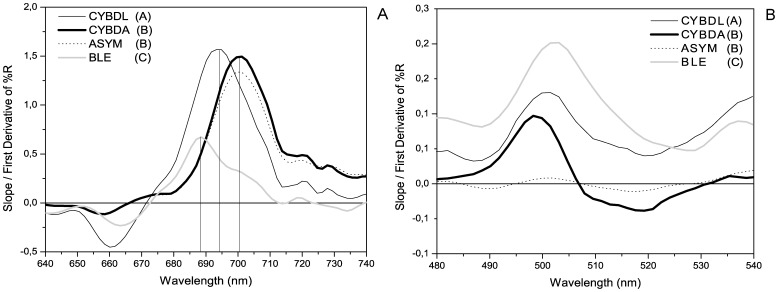
Spectral Indices (A) First derivative of percent reflectance (%R) in the red edge region. Vertical lines denote the inflection point of the red edge position between 640 and 740(Kruskal Wallis Analysis: H = 18.53. p<0.0005). (B) First derivative of percent reflectance (%R) in the blue edge region (SD_B_). Statistically significant differences in sum of the first derivative between 490 and 530 nm (Kruskal Wallis analysis: H = 20.21, p<0.0005). Different letters in parentheses in both (A) and (B) denote significant differences between colony condition (Mann-Whitney pairwise comparison results, α = 0.05).

The two remote sensing indices that demonstrated the ability to separate colony conditions by first derivatives of %R were the red edge position and SD_B_. There was a statistically significant effect of colony condition on median red edge position (KW results: H = 18.53, p<0.0005). Pairwise MW comparisons (α = 0.05) demonstrated significant differences in the red edge position for CYBDL at 694 nm and BLE at 689 nm. Conditions CYBDA and ASYM both had the same red edge position at 701 nm (Figure 4a). All colony conditions were separable by the SD_B_ index (Figure 4B) (KW results: H = 20.21, p<0.0005; MW comparisons: α = 0.05).

**Figure 5:**
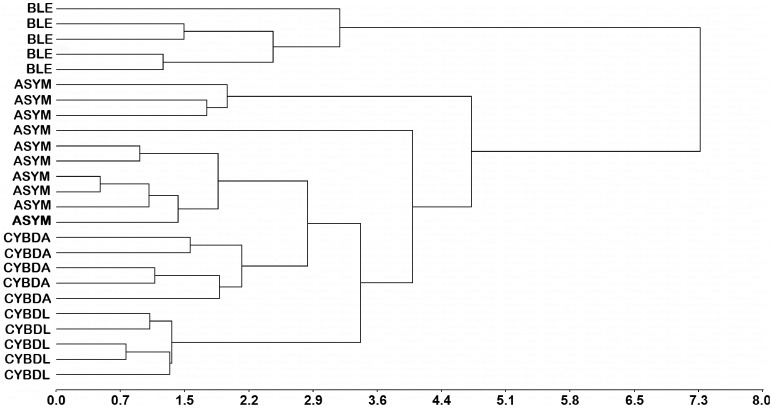
Weighted average linkage cluster analysis with Euclidian distances. Cophenetic correlation coefficient  =  0.87.

Finally, the ability to separate colony condition by the entire remote sensing reflectance spectra in the visible region is presented in the cluster analysis tree (Figure 5), which demonstrates the separation of CYBDL, CYBDA, BLE, and ASYM conditions into separate clades with a cophenetic correlation coefficient of 0.87. While the disease conditions separate into discrete clades, 6 ASYM colonies formed one core cluster with 4 outlying colonies.

## Discussion

In an effort to expand the array of tools available for investigating and monitoring coral disease, the spectral features associated with *O. faveolata* affected by CYBD were characterized for the first time. The method of hyperspectral remote sensing was used to measure reflectance of light in the visible spectrum from living coral tissue affected by different disease states (CYBDL, CYBDA, BLE and ASYM). Reflectance spectra were studied using derivative analysis, spectral indices and cluster analysis. The results demonstrate that these methods can be used to identify spectral signatures that are unique to each coral disease condition and separate samples into appropriate disease state. Similar methods have been used in the field of coral biology [36]–[40], as well as agriculture and forestry [77]–[79], but this is the first report of the application of hyperspectral remote sensing to the investigation of infectious coral diseases. The main objective of the present study was not to develop a universal tool for coral disease diagnosis, but rather introduce a novel approach to monitoring coral diseases. By measuring spectral reflectance *ex situ* under controlled laboratory conditions, variability between sample measurements was reduced. However, natural variability in wild coral populations would likely limit the immediate application of this method to *in situ* monitoring efforts. Factors that could limit its application include, for example: phenotypic plasticity in host and symbiont pigment compositions [80]–[81]; phenotypic plasticity in immune responses to disease [82]; and physical parameters of the water column, such as light attenuation [83]. It was not within the scope of this investigation to model the effects of interacting biological factors and oceanographic parameters on reflectance data. This is a topic that has been investigated extensively in the field of remote sensing of coral reef environments [46], [76].

Despite a limited sample size, the present study was able to reveal statistically significant patterns in spectral reflectance unique to each sample condition. Genotyping was not conducted to assess whether samples were from clonal colonies. However, asexual reproduction by fragmentation is not common in massive colonies of *O. faveolata*. Population genetic studies of the same species and sampling site, Media Luna, have revealed low levels of clonality (3.5%) [84]. Therefore, each sample was assumed to represent a unique genotype. This assumption and the sample size are comparable to studies published to date on coral health [12], [20]–[24]. Small sample sizes are often the result of the unique circumstances presented by disease outbreaks, which was especially the case in the present study. CYBD has resulted in drastic declines in coral cover in Puerto Rico [54]–[55], which makes it difficult to find large numbers of colonies of each condition. In the present study, samples were collected from Media Luna, which is mid-shelf reef in La Parguera that has a relatively large number of ASYM and CYBD host colonies [85].

Despite the limitations mentioned above, the results presented provide significant evidence that hyperspectral remote sensing is a suitable means to monitor coral disease and health. Cluster analysis of the overall hyperspectral reflectance spectra demonstrated that this method can be used to separate diseased and asymptomatic *O. faveolata* tissue into discrete groups (CYBDA, CYBDL, BLE, ASYM) (Figure 5). Analysis of the data using spectral indices was also able to discriminate between sample conditions. The red edge position, which is dependent on photosynthetic pigments in *Symbiodinium* [39], was significantly different for CYBDL and BLE colonies (Figure 4). From a disease diagnosis perspective, the red edge position did not improve visual diagnoses by differentiating between the conditions, CYBDA and ASYM, which are indistinguishable in the field. However, the results provide an interesting perspective on the use of *in vivo* spectroscopy to study coral disease etiology. Thermal-induced BLE and CYBDL are both diseases of the host and photosynthetic endosymbiont, *Symbiodinium*. Although BLE and CYBD symptoms are similar (i.e. paling of coral tissue), their etiologies quite different [54], [56], [86]–[88]. The results support the established CYBDA etiology that *Symbiodinium* cells remain unaffected by the causative agents by demonstrating that shifts in the red edge position do not occur relative to ASYM tissue. Similarly, the different effects of CYBD and BLE on *Symbiodinium* are demonstrated by differential shifts in CYBDL and BLE red edge positions relative to ASYM. One challenge in applying the red edge index to *in situ* spectroscopy is the rapid attenuation of red light through the water column [39]. However, this may be resolved simply by integrating the use of artificial, full-spectrum sources of illumination. The remote sensing index SD_B_ has been used in various spectral studies of crop plants [30], [77]. In the present investigation, the SD_B_ index was able to discriminate between all of the disease conditions (Figure 4B). Derivative spectra between 490 and 530 nm are used in the SD_B_ analysis, and therefore, the diagnostic power of this index is likely dependent on pigment signatures that are amplified in this region. Immune related pigments, such as green fluorescent proteins (GFP), have emission peaks that span the same wavelengths used to derive the SD_B_ index. The diagnostic ability of the SD_B_ index may be related to the fact that GFPs are indicators of disease susceptibility and immunocompetence in a variety of coral species [23]. While visual diagnosis of BLE and CYBDL is straight-forward for trained personnel in the field, to date significant differences in ASYM and CYBDA have only been revealed using molecular biomarkers [17]. The ability to distinguish between these conditions by using cluster analysis and spectral indices suggests that *in vivo* spectroscopy can be used to enhance the accuracy of diagnoses. Since most coral diseases are routinely diagnosed by visual symptoms of tissue mortality [8], the power to detect spectral markers of disease before symptoms become visible could drastically reduce response times, and in turn, improve the efficacy of established monitoring programs. For example, it can take several months after the first report of a disease outbreak to acquire results on coral health using molecular biomarkers. Long processing times and high costs are associated with the preservation, transport, and analysis of samples in specialized molecular biology laboratories. Alternatively, using the spectroscopic approach detailed in the present study, direct measurements were taken within 24 h of sample collection. Interpretation of the data can be completed within one week by using an established set of analyses. Analyses can be optimized further to reduce response time through the integration of automated computer algorithms, similar to methods used in the field of food quality monitoring [89]. Rapid response times are especially necessary during outbreaks of the most virulent coral diseases, which can lead to widespread coral mortality in only a few months after the first appearance of lesions [90]. Research to expand the use of hyperspectral reflectance in coral disease monitoring should focus on establishing the spectral characteristics of a greater diversity of coral species and diseases. Additionally, as a larger base of data on the spectral features of diseased corals becomes available, analyses using more robust multivariate statistical methods (e.g. step-wise discriminant analysis, principle component analysis) and coral disease-specific spectral indices can be developed.

Recent investigations have demonstrated that pigments play an essential role in regulating coral innate immunity, among which melanin and fluorescent proteins are the most well characterized (Table 1). The synthesis and accumulation of these pigments are essential for regulating redox potential in coral cells [25]. Apart from the diagnostic power that spectroscopy offers, this method can also be used to explore the pigment composition in living coral tissues and the role of pigments in coral disease etiology. The present study used derivative analysis to highlight spectral signatures that significantly contributed to differences between disease conditions (Table 3). A statistically significant spectral signature for green fluorescent protein (GFP) at 505 nm was detected in the diseased samples (BLE, CYBDL, and CYBDA, Figure 5), further supporting the aforementioned hypothesis that GFP contributes to the diagnostic power of the SD_B_ index. Derivative peaks at this wavelength were able to discriminate ASYM samples from all other colony conditions (Table 3). The magnitudes, from least to greatest, of the first derivative peaks in the region of GFP emission (Figure 4b) for each condition were ASYM, CYBDA, CYBDL and BLE, respectively. Since the physiological states of BLE, CYBDA, CYBDL lead to the overproduction of ROS, the results support the generalized ROS-scavenging role of GFP in diseased coral tissues [17], [84]. Melanin pigments have a nearly linear reflectance spectrum making their contribution to differences between disease conditions difficult to extract by derivative analysis in the present investigation [91]. However, melanin synthesis pathways are known to be activated in *O. faveolata* affected by CYBD [17]. The overall reduced albedo in CYBDA tissues compared to ASYM tissues may be a result of increased synthesis of this immune-regulated pigment (Figure 2).

Bleaching and CYBD are characterized by the expulsion or lysis of the photosynthetic endosymbiont, *Symbiodinium*, respectively [86]–[88]. Therefore, it was expected that significant spectral changes in these conditions would be due to shifts in the composition of *Symbiodinium* pigments present in the coral cells. *Symbiodinium* pigments that contributed putatively to spectral differences in disease conditions of the present investigation include carotenoids, chlorophyll, xanthophyll, fucoxanthin, diadinoxanthin, phycobilin, phycocyanin, and peridinin (Table 3). These results are supported by a report of similar changes in the composition of *Symbiodinium* pigments in corals exposed to thermal stress and bleaching [92].

Although bacterial causative agents and characteristic symptoms of CYBD are well established, the roles of *Symbiodinium* pigments in CYBD etiology remain unclear [8], [59], [87]. A key characteristic of CYBD is the persistence of *Symbiodinium* in infected tissue despite widespread lysis caused by bacterial pathogens [88]. This results in partially bleached lesion tissue, which has a characteristic yellow pigmentation (Figure 1). Therefore, unique patterns of spectral reflectance were expected for CYBDL and BLE samples. This hypothesis was supported by the aforementioned shifts in the red edge position (Figure 4A), and results of the derivative analysis, which demonstrated statistically significant differences between CYBDL and BLE in pairwise MW comparisons (Table 3). Putative pigments that contributed to unique spectral signatures in CYBDL samples were α-carotenoid (423 to 471 nm), fucoxanthin (526 to 553 nm), peridinin (540 nm), phycocyanin (617 to 640 nm). The role of these *Symbiodinium* pigments in disease etiology cannot be elucidated from the results of the present study. However, these putative associations can provide a framework for future investigations. For example, one particularly interesting association that has not been tested to date is the contribution of carotenoids to the characteristic yellow appearance of CYBDL. The synthesis of carotenoids is widely documented in eukaryotic phototrophs, including *Symbiodinium. Symbiodinium*-derived carotenoids likely contribute to the physiological requirement of this pigment in corals [93]. While invertebrates cannot synthesize carotenoids directly, they have evolved mechanisms of obtaining this pigment through digestion, which is regulated by a group of carotenoid-binding proteins [94]. Fluctuation of carotenoids in bleached corals has been documented, yet the mechanisms that regulate the presence of these pigments in corals remain unclear [95]. High concentrations of carotenoids are known to contribute yellow coloration in a wide variety of organisms. Therefore, it can be hypothesized that the yellow color of CYBD lesion tissue may arise from a high retention of this pigment. This hypothesis is supported by current knowledge about the regulation of carotenoids as antioxidants in the invertebrate innate immune system [96]. Antioxidant production and ROS detoxification are processes that underpin coral immune responses to CYBD [20], [21]. Therefore, the accumulation of carotenoids in CYBDL tissue could be the result of cooperative interactions between the coral immune system and endosymbionts that reduce ROS-induced cellular damage.

In summary, the results and design of the present study are immediately applicable to monitoring coral disease in captive populations of corals in mariculture, aquaculture and aquaria. There have been reports of disease outbreaks in these areas, which have the potential to greatly impact the ultimate goal of such activities: the restoration of degraded coral reefs, and the commercial sale of sustainably-grown corals for research and education [65]–[69]. The concept of using spectral data to investigate coral health status can be further developed for non-invasive, *in situ* applications. For example, diver-operated underwater spectral radiometers and cameras can detect high resolution reflectance patterns *in situ* from subjects illuminated artificially with full-spectrum visible light [37], [97], [98]. This would improve *in situ* coral disease diagnoses and reduce the need for destructive sampling methods, which are commonly used to assess coral health with molecular and cellular biomarkers. Most notably, the application of hyperspectral sensing to coral health and disease monitoring has the potential to reduce response times to virulent disease outbreaks and improve the success of mitigation strategies.
